# ﻿Four new species of *Entoloma* subgen. *Cubospora* (Entolomataceae, Agaricales) from Yunnan Province, China

**DOI:** 10.3897/mycokeys.124.171541

**Published:** 2025-11-11

**Authors:** Ze-Wei Liu, Jing Li, Zhu L. Yang, Yang-Yang Cui

**Affiliations:** 1 Key Laboratory of Phytochemistry and Natural Medicines, Kunming Institute of Botany, Chinese Academy of Sciences, Kunming 650201, China Key Laboratory of Phytochemistry and Natural Medicines, Chinese Academy of Sciences Kunming China; 2 Yunnan Key Laboratory for Fungal Diversity and Green Development, Kunming 650201, China Yunnan Key Laboratory for Fungal Diversity and Green Development Kunming China; 3 University of Chinese Academy of Sciences, Beijing 100049, China University of Chinese Academy of Sciences Beijing China; 4 Faculty of Geography, Yunnan Normal University, Kunming 650500, China Yunnan Normal University Kunming China

**Keywords:** Cuboid spores, new taxa, phylogeny, subtropical, taxonomy

## Abstract

Species of *Entoloma* are exceptionally diverse and widely distributed, with cuboid-spored members occurring mainly in subtropical to tropical regions. These cuboid-spored taxa are primarily classified into two subgenera, E. subg. Cuboeccilia and E. subg. Cubospora. During surveys conducted in Yunnan Province, a representative subtropical region of China, four new species belonging to E. subg. Cubospora were identified through multigene phylogenetic analyses (ITS, LSU, *rpb2*, and *tef1*) combined with detailed morphological observations. *Entoloma acutiflavum* is distinguished by its yellow basidiomata, papillate pileus, heterogeneous lamellar edge, and the presence of clamp connections. *Entoloma bichromum* can be recognized by a centrally papillate pileus, serrate pileal and lamellar margins, and clavate to cylindrical pleurocystidia that are flexuous, furcate, or occasionally ventricose. *Entoloma guttuliferum* is characterized by its yellowish-white to white papillate pileus, clavate to elongated clavate terminal cells in both the pileipellis and stipitipellis, and pigmented cystidia, lamellar trama, pileipellis, and stipitipellis. *Entoloma rufosquamulosum* is identified by its reddish-brown to grayish-violet squamose pileus, with reddish-brown pigments present in the upper hyphal layer and terminal cells of the pileipellis. Detailed descriptions, line drawings, habitat photographs, SEM micrographs, and comparisons with morphologically and phylogenetically related taxa are provided. A key to the species of Entoloma subg. Cubospora from China is also included.

## ﻿Introduction

Species of *Entoloma* are globally distributed and abundant in both temperate and tropical regions, with some species even extending into alpine and frigid zones (Largent 1977, 1994; Romagnesi and Gilles 1979; Horak 1980, 2008; Baroni 1981; Noordeloos 1988, 1992, 2004; Gates and Noordeloos 2007; Noordeloos and Hausknecht 2007; Gates et al. 2009; Noordeloos and Gates 2009, 2012; He 2012; Karstedt et al. 2024). To date, more than 2,000 species have been described. Despite their remarkable morphological diversity, all *Entoloma* species produce pink spore prints, and their basidiospores are angular in all orientations (Co-David et al. 2009; Noordeloos et al. 2018).

Among angular-spored *Entoloma* species, those with cuboid basidiospores are relatively easy to recognize. Historically, however, taxa with this character were inconsistently classified into different subgenera or sections based solely on morphology—such as sect. Staurospora within subg. Nolanea or subg. Inocephalus (Noordeloos 1992; Largent 1994). To clarify their phylogenetic positions, Karstedt et al. (2019) conducted extensive sampling and demonstrated that these taxa form two distinct subgenera, subg. Cuboeccilia and subg. Cubospora, as supported by both molecular data and morphological features of basidiomata and cystidia.

In recent decades, numerous cuboid-spored species have been discovered across tropical and subtropical regions. To date, approximately 190 such species have been reported worldwide, with only a few occurring in temperate zones (Horak 1976, 1977; Romagnesi and Gilles 1979; He et al. 2015a; Karstedt et al. 2019, 2024; Reschke et al. 2022a; Morozova and Pham 2023; Chen et al. 2024; Sato et al. 2025). In China, several new cuboid-spored species have been described from subtropical provinces such as Guangdong, Fujian, and Jiangxi (He et al. 2015a; Chen et al. 2024). Through field explorations in Yunnan Province, we discovered four novel species belonging to E. subg. Cubospora. These taxa exhibit distinct morphological characteristics and phylogenetic differentiation from all previously known species, as demonstrated by both morphological and multilocus analyses.

## ﻿Materials and methods

### ﻿Morphological studies

Specimens collected between 2010 and 2023 in Yunnan Province, China, were deposited in the Herbarium of the Kunming Institute of Botany, Chinese Academy of Sciences (KUN-HKAS). Macroscopic characters were recorded and photographed from fresh basidiomata in the field. Color designations followed Kornerup and Wanscher (1978). Microscopic characters were examined under a ZEISS Axio Scope A1 light microscope. Sections of dried material were rehydrated in 5% KOH or H_2_O and stained with 1% (w/v) aqueous Congo red when necessary. Melzer’s reagent was used to test for spore amyloidity (Clémençon et al. 2004; Horak 2005). Line drawings were prepared freehand under a phase-contrast objective (1000×). At least 20 basidiospores were measured, and data are presented as (a/b/c) (d)e–***f***–g(h) × (i)j–***k***–l(m) μm [Q = (n)o–p(q), **Q** = r ± s]. Here, a–c indicates that *a* basidiospores were measured from *b* basidiomata of *c* specimens. Parameters *d* and *h* represent the minimum and maximum (5% extreme) values of length; *e–g* denote the central 90% range. The same applies to the width parameters (*i–m*) and the *Q* values (*n–q*). The mean length (*f*), width (*k*), and *Q* ratio (*r*), together with the standard deviation of *Q* (*s*), are also provided (Liu et al. 2022; Feng et al. 2025). Basidiospores were further examined using a Zeiss Sigma 300 scanning electron microscope (Oberkochen, Germany).

### ﻿DNA extraction, PCR amplification, and DNA sequencing

Genomic DNA was extracted from dried basidiomata using the Ezup Column Fungi Genomic DNA Purification Kit (Sangon Biotech, Shanghai, China). Four nuclear loci were amplified: the internal transcribed spacer (ITS), the large subunit ribosomal RNA (LSU), RNA polymerase II subunit 2 (*rpb2*), and translation elongation factor 1-alpha (*tef1*). PCR reactions (25 μL) contained 12.5 μL of 2× Taq Master Mix, 8.5 μL of nuclease-free water, 2 μL of primers, and 2 μL of DNA template. Primer pairs were ITS1F/ITS4 and LR0R/LR5 for ITS and LSU (Vilgalys and Hester 1990; Gardes and Bruns 1993), *rpb2*-i6f/*rpb2*-RhoR1 for *rpb2*, and EF1-983ClitoF1/EF1-1953ClitoR1 or EF1-983ClitoF4/EF1-1953ClitoR4 for *tef1* (Kluting et al. 2014; Zhong 2019; Jian 2020). PCR conditions were as follows: for ITS, initial denaturation at 94 °C for 3 min; 35 cycles of 94 °C for 30 s, 53 °C for 40 s, and 72 °C for 1 min; and a final extension at 72 °C for 8 min. For LSU, the protocol was 94 °C for 3 min; 35 cycles of 94 °C for 30 s, 48 °C for 40 s, and 72 °C for 90 s; and a final extension at 72 °C for 8 min. Protocols for *rpb2* and *tef1* followed Zhong (2019) and Jian (2020), respectively. All sequencing and cloning were conducted by Sangon Biotech (Shanghai, China).

### ﻿Alignment and phylogenetic analysis

Sequences of Entoloma subg. Cubospora were downloaded from GenBank and combined with sequences newly generated in this study. Three representative species from E. subg. Nolanea were selected as outgroups. The four gene regions (ITS, LSU, *rpb2*, and *tef1*) were aligned separately using MAFFT v7 (Katoh and Standley 2013), manually adjusted in BioEdit v7.2.5 (Hall 1999; Thompson et al. 1997), and concatenated into a single matrix. MODELTEST v2.3 was used to determine the best-fit substitution model for each gene based on the Akaike Information Criterion (Posada and Crandall 1998). Bayesian inference (BI) was performed using MrBayes implemented in PhyloSuite v1.2.3, with two independent runs of 60 million generations, sampling every 10,000 generations. The analysis was terminated when the average standard deviation of split frequencies dropped below 0.01, and the first 25% of trees were discarded as burn-in using the “sump” and “sumt” commands (Zhang et al. 2020). Maximum likelihood (ML) analysis was conducted using RAxMLGUI v2.0.5 with 1,000 bootstrap replicates under the GTRGAMMAI model (Edler et al. 2021).

## ﻿Results

### ﻿Phylogenetic relationships

**Figure 1.**
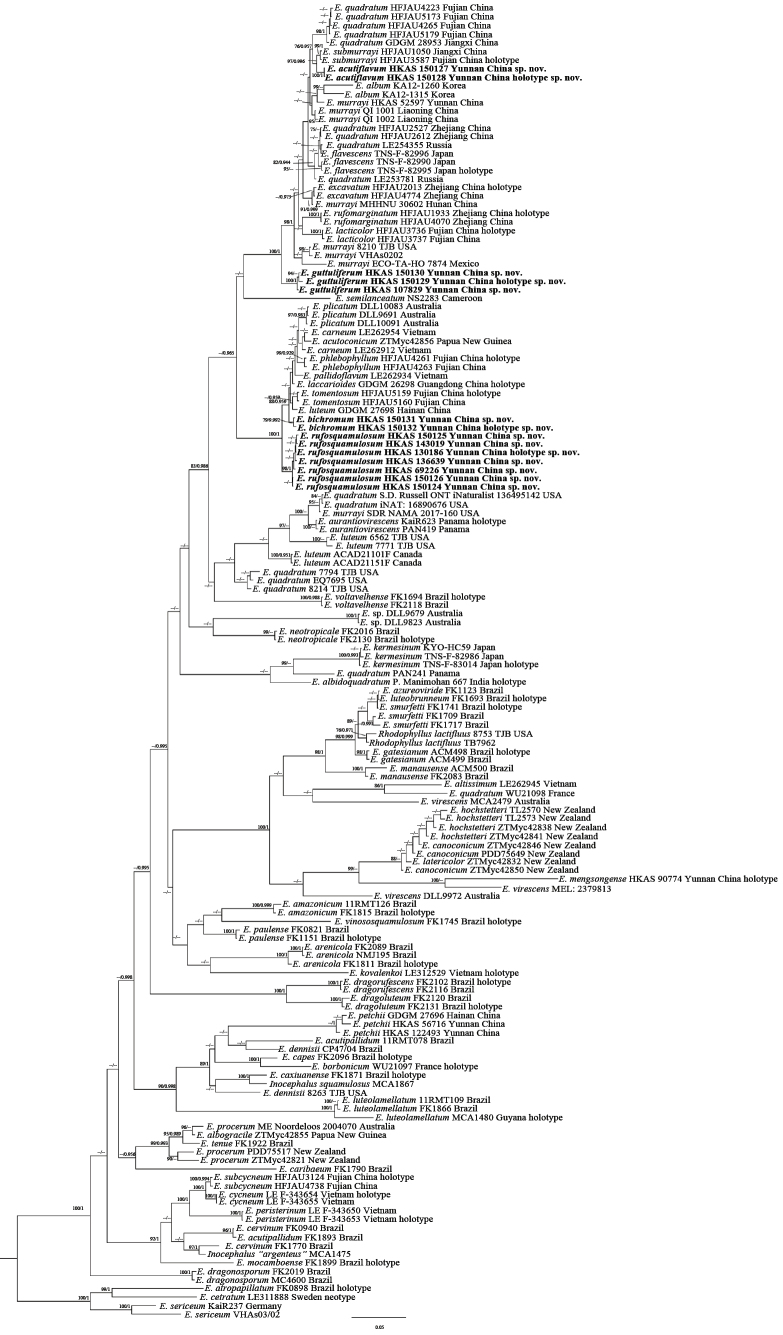
Bayesian inference analysis of Entoloma subg. Cubospora based on ITS, LSU, *rpb2*, and *tef1* sequence data. *E. atropapillatum*, *E. cetratum*, and *E. sericeum* serve as outgroups. Bootstrap values (BS) from maximum likelihood ≥ 75 and Bayesian posterior probabilities (BPP) ≥ 0.95 are shown on each branch (BS/BPP). The new species are marked in bold.

The combined dataset included 358 sequences representing 154 specimens and 68 species, comprising 53 newly generated and 305 retrieved from GenBank (Table 1). The concatenated alignment consisted of 3,254 characters. The best-fit models were GTR+I+G for ITS and LSU, SYM+I+G for *rpb2*, and HKY+I+G for *tef1*. After 60 million generations, the BI analysis reached convergence (average split frequency < 0.01). The BI and ML topologies were highly congruent; therefore, the BI tree is presented (Fig. 1). Entoloma subg. Cubospora formed a robust monophyletic clade (BS/Bayesian posterior probability [BPP] = 100/1.00). The four new taxa—*E. acutiflavum*, *E. bichromum*, *E. guttuliferum*, and *E. rufosquamulosum*—each formed well-supported, independent lineages within E. subg. Cubospora. No closely related species were identified for *E. bichromum*, *E. guttuliferum*, or *E. rufosquamulosum*, whereas *E. acutiflavum* was recovered as sister to *E. submurrayi*.

**Table 1.** Specimens used in phylogenetic analysis and GenBank accession numbers.

No.	Species	Specimen voucher	GenBank accession numbers				Locality	Reference
			ITS	LSU	*rpb2*	*tef1*		
**1.**	***Entoloma acutiflavum***	**HKAS 150127**	**PX269826**	**PX269836**	**PX255550**	**PX255527**	**China**	**in this study**
**2.**	***E. acutiflavum***	**HKAS 150128, holotype**	**PX269825**	**PX269837**	**PX255549**	**PX255526**	**China**	**in this study**
3.	*E. acutipallidum*	11RMT078	–	MW624792	MW624730	–	Brazil	Karstedt et al. 2024
4.	*E. acutipallidum*	FK1893	–	MG018325	–	MH190147	Brazil	Karstedt et al. 2019
5.	*E. acutoconicum*	ZTMyc42856	–	MW624791	–	–	Papua New Guinea	Karstedt et al. 2024
6.	*E. albidoquadratum*	P. Manimohan 667, holotype	–	GQ289151	GQ289223	–	India	Co-David et al. 2009
7.	*E. albogracile*	ZTMyc42855	–	MH190207	–	–	Papua New Guinea	Karstedt et al. 2019
8.	*E. album*	KA12-1260	KR673507	–	–	–	Korea	Kim et al. 2015
9.	*E. album*	KA12-1315	KR673546	–	–	–	Korea	Kim et al. 2015
10.	*E. altissimum*	LE262945	MF476912	MW624793	MW624731	–	Vietnam	Karstedt et al. 2024
11.	*E. amazonicum*	11RMT126	–	MW624794	MW624732	MW624839	Brazil	Karstedt et al. 2024
12.	*E. amazonicum*	FK1815, holotype	–	MW624795	–	–	Brazil	Karstedt et al. 2024
13.	*E. arenicola*	FK1811, holotype	–	MW624796	MW624733	–	Brazil	Karstedt et al. 2024
14.	*E. arenicola*	FK2089	–	MW624797	MW624734	MW624840	Brazil	Karstedt et al. 2024
15.	*E. arenicola*	NMJ195	–	MW624799	MW624735	MW624841	Brazil	Karstedt et al. 2024
16.	*E. atropapillatum*	FK0898, holotype	KF679354	KF738940	MH190107	MH190137	Brazil	Karstedt et al. 2019, 2020
17.	*E. aurantiovirescens*	KaiR623, holotype	MZ611665	–	–	–	Panama	Reschke et al. 2022a
18.	*E. aurantiovirescens*	PAN419	MZ611691	–	–	–	Panama	Reschke et al. 2022a
19.	*E. azureoviride*	FK1123	–	MW624830	MW624752	MW624855	Brazil	Karstedt et al. 2024
**20.**	***E. bichromum***	**HKAS 150131**	**PX269814**	**PX269835**	**PX255539**	**PX255531**	**China**	**in this study**
**21.**	***E. bichromum***	**HKAS 150132, holotype**	**PX269813**	**PX269834**	**PX255538**	**PX255530**	**China**	**in this study**
22.	*E. borbonicum*	WU21097, holotype	–	MH190198	MH190131	MH190166	France	Karstedt et al. 2019
23.	*E. canoconicum*	PDD75649	–	MW624800	–	–	New Zealand	Karstedt et al. 2024
24.	*E. canoconicum*	ZTMyc42846	–	MW624801	–	–	New Zealand	Karstedt et al. 2024
25.	*E. canoconicum*	ZTMyc42850	–	MW624802	MW624736	–	New Zealand	Karstedt et al. 2024
26.	*E. capes*	FK2096, holotype	–	MW624803	MW624737	–	Brazil	Karstedt et al. 2024
27.	*E. caribaeum*	FK1790	–	MH190214	MH190114	MH190146	Brazil	Karstedt et al. 2019
28.	*E. carneum*	LE262912	–	MH190181	MH190119	MH190152	Vietnam	Karstedt et al. 2019
29.	*E. carneum*	LE262954	–	MH190184	MH190121	–	Vietnam	Karstedt et al. 2019
30.	*E. caxiuanense*	FK1871, holotype	–	MW624804	–	–	Brazil	Karstedt et al. 2024
31.	*E. cervinum*	FK0940, holotype	–	–	MG018332	MH190138	Brazil	Karstedt et al. 2019
32.	*E. cervinum*	FK1770	–	MW624805	–	–	Brazil	Karstedt et al. 2024
33.	*E. cetratum*	LE311888, neotype	OL338280	–	OL405215	OL405538	Sweden	Reschke et al. 2022b
34.	*E. cycneum*	LE F-343654, holotype	OQ779461	OQ804518	–	OQ779183	Vietnam	Morozova and Pham 2023
35.	*E. cycneum*	LE F-343655	OQ779463	OQ804519	–	OQ779182	Vietnam	Morozova and Pham 2023
36.	*E. dennisii*	8263 TJB	–	MH190195	MH190128	MH190164	USA	Karstedt et al. 2019
37.	*E. dennisii*	CP47/04	–	MH190210	–	–	Brazil	Karstedt et al. 2019
38.	*E. dragoluteum*	FK2120	–	MW624807	MW624739	MW624842	Brazil	Karstedt et al. 2024
39.	*E. dragoluteum*	FK2131, holotype	–	MW624806	MW624738	–	Brazil	Karstedt et al. 2024
40.	*E. dragonosporm*	FK2019	–	MH190179	MG018336	MH190150	Brazil	Karstedt et al. 2019
41.	*E. dragonosporm*	MC4600	–	MH190186	MH190122	MH190156	Brazil	Karstedt et al. 2019
42.	*E. dragorufescens*	FK2102, holotype	–	MW624810	MW624740	MW624843	Brazil	Karstedt et al. 2024
43.	*E. dragorufescens*	FK2116	–	MW624811	MW624741	–	Brazil	Karstedt et al. 2024
44.	*E. excavatum*	HFJAU2013, holotype	PP796416	PP789602	–	–	China	Chen et al. 2024
45.	*E. excavatum*	HFJAU4774	PP796431	PP789614	–	–	China	Chen et al. 2024
46.	*E. flavescens*	TNS-F-82990	ITS1:LC786658 ITS2:LC786709	KF723693	KF723647	–	Japan	Sato et al. 2025
47.	*E. flavescens*	TNS-F-82995, holotype	ITS1:LC786663 ITS2:LC786714	KF723691	KF723645	–	Japan	Sato et al. 2025
48.	*E. flavescens*	TNS-F-82996	ITS1:LC786664 ITS2:LC786715	KF723692	KF723646	–	Japan	Sato et al. 2025
49.	*E. gatesianum*	ACM498, holotype	–	MW624812	MW624742	MW624844	Brazil	Karstedt et al. 2024
50.	*E. gatesianum*	ACM499	–	MW624813	–	–	Brazil	Karstedt et al. 2024
**51.**	***E. guttuliferum***	**HKAS 107829**	**PX269816**	**PX269840**	**PX255541**	–	**China**	**in this study**
**52.**	***E. guttuliferum***	**HKAS 150129, holotype**	**PX269815**	**PX269838**	**PX255540**	**PX255528**	**China**	**in this study**
**53.**	***E. guttuliferum***	**HKAS 150130**	**PX269817**	**PX269839**	**PX255542**	**PX255529**	**China**	**in this study**
54.	*E. hochstetteri*	TL2570	KP191939	KP191755	–	–	New Zealand	Unpublished
55.	*E. hochstetteri*	TL2573	KP191941	KP191758	–	–	New Zealand	Unpublished
56.	*E. hochstetteri*	ZTMyc42838	–	MW624814	–	–	New Zealand	Karstedt et al. 2024
57.	*E. hochstetteri*	ZTMyc42841	–	OP836300	–	–	New Zealand	Karstedt et al. 2024
58.	*E. kermesinum*	KYO-HC59	ITS1:LC786696 ITS2:LC786749	–	–	–	Japan	Sato et al. 2025
59.	*E. kermesinum*	TNS-F-82986	ITS1:LC786654 ITS2:LC786705	–	–	–	Japan	Sato et al. 2025
60.	*E. kermesinum*	TNS-F-83014, holotype	ITS1:LC786677 ITS2:LC786729	–	–	–	Japan	Sato et al. 2025
61.	*E. kovalenkoi*	LE312529, holotype	OK257210	OK257207	–	OK256169	Vietnam	Crous et al. 2021
62.	*E. laccarioides*	GDGM 26298	–	JQ993091	–	–	China	He et al. 2013
63.	*E. lacticolor*	HFJAU3736, holotype	OR683793	OR687490	OR738710	OR699451	China	Chen et al. 2024
64.	*E. lacticolor*	HFJAU3737	OR683794	OR687491	OR738711	OR699452	China	Chen et al. 2024
65.	*E. latericolor*	ZTMyc42832	–	MW624815	–	MW624845	New Zealand	Karstedt et al. 2024
66.	*E. luteobrunneum*	FK1693, holotype	–	MW624816	–	MW624846	Brazil	Karstedt et al. 2024
67.	*E. luteolamellatum*	11RMT109	–	MH190170	MH190105	–	Brazil	Karstedt et al. 2019
68.	*E. luteolamellatum*	FK1866	–	MW624817	MW624743	–	Brazil	Karstedt et al. 2024
69.	*E. luteolamellatum*	MCA1480 holotype	–	MH190213	MH190135	MG702644	Guyana	Karstedt et al. 2019
**70.**	*E. luteum*	6562 TJB	–	KR869944	–	–	USA	Largent et al. 2016
**71.**	*E. luteum*	7771 TJB	–	MH190212	MH190125	MH190161	USA	Karstedt et al. 2019
**72.**	*E. luteum*	ACAD21101F	OM716995	–	–	–	Canada	Unpublished
**73.**	*E. luteum*	ACAD21151F	ON412813	–	–	–	Canada	Unpublished
**74.**	*E. luteum*	GDGM 27698	JQ281486	JQ320121	–	–	China	He et al. 2012, 2013
75.	*E. manausense*	ACM500	–	MW624818	MW624744	MW624847	Brazil	Karstedt et al. 2024
76.	*E. manausense*	FK2083	–	MW624819	MW624745	–	Brazil	Karstedt et al. 2024
**77.**	*E. mengsongense*	HKAS90774, holotype	KU131556	–	–	–	China	Ediriweera et al. 2017
78.	*E. mocamboense*	FK1899, holotype	–	MW624820	–	MW624848	Brazil	Karstedt et al. 2024
79.	*E. murrayi*	8210 TJB	–	MH190193	MH190127	–	USA	Karstedt et al. 2019
**80.**	*E. murrayi*	ECO-TA-HO 7874	MF156254	–	–	–	Mexico	Hernández 2017
**81.**	*E. murrayi*	HKAS 52597	–	KJ648469	–	–	China	He 2012
**82.**	*E. murrayi*	MHHNU 30602	MK250917	–	–	–	China	Chen and Zhang 2019
**83.**	*E. murrayi*	QI 1002	KJ658968	JQ993089	JQ993082	–	China	He et al. 2013
**84.**	*E. murrayi*	QI 1001	KJ658967	JQ993090	JQ993081	–	China	He et al. 2013
**85.**	*E. murrayi*	SDR NAMA 2017-160	MK575459	–	–	–	USA	Unpublished
**86.**	*E. murrayi*	VHAs0202	–	GU384620	GU384637	–		Baroni et al. 2011
87.	*E. neotropicale*	FK2016	–	MW624822	MW624746	MW624850	Brazil	Karstedt et al. 2024
88.	*E. neotropicale*	FK2130, holotype	–	MW624825	MW624748	MW624853	Brazil	Karstedt et al. 2024
89.	*E. pallidoflavum*	LE262934	OQ779469	MH190183	MH259314	MH190155	Vietnam	Karstedt et al. 2019
90.	*E. paulense*	FK0821	–	MW624826	MW624749	–	Brazil	Karstedt et al. 2024
91.	*E. paulense*	FK1151, holotype	–	MW624827	MW624750	–	Brazil	Karstedt et al. 2024
92.	*E. peristerinum*	LE F-343650	OQ779467	OQ804524	–	OQ779186	Vietnam	Morozova and Pham 2023
93.	*E. peristerinum*	LE F-343653, holotype	OQ779466	OQ804522	–	OQ779188	Vietnam	Morozova and Pham 2023
94.	*E. petchii*	GDGM 27696	–	JX992853	–	–	China	He 2012
95.	*E. petchii*	HKAS 56716	JQ281485	JQ320120	–	–	China	He et al. 2012
96.	*E. petchii*	HKAS 122493	ON794324	–	–	–	China	Unpublished
97.	*E. phlebophyllum*	HFJAU4261, holotype	OR827447	OR825714	OR827308	OR827307	China	Chen et al. 2024
98.	*E. phlebophyllum*	HFJAU4263	OR827448	–	–	–	China	Chen et al. 2024
99.	*E. plicatum*	DLL9691	–	JQ624610	JQ624617	–	Australia	Largent et al. 2013
100.	*E. plicatum*	DLL10083	–	JQ624612	JQ624619	MG702626	Australia	Largent et al. 2013; Karstedt et al. 2019
101.	*E. plicatum*	DLL10091	–	JQ624613	JQ624620	–	Australia	Largent et al. 2013
102.	*E. procerum*	ME Noordeloos 2004070	–	GQ289183	GQ289254	–	Australia	Co-David et al. 2009
103.	*E. procerum*	PDD75517	–	MH190189	–	–	New Zealand	Karstedt et al. 2019
104.	*E. procerum*	ZTMyc42821	–	MH190201	–	MH190167	New Zealand	Karstedt et al. 2019
105.	*E. quadratum*	7794 TJB	–	MH190192	MH190126	–	USA	Karstedt et al. 2019
106.	*E. quadratum*	8214 TJB	–	MH190194	–	MH190162	USA	Karstedt et al. 2019
107.	*E. quadratum*	EQ7695	–	AF261303	–	–	USA	Moncalvo et al. 2002
108.	*E. quadratum*	GDGM 28953	–	KJ648471	KP226183	–	China	He et al. 2015a, b
109.	*E. quadratum*	HFJAU2527	PP796418	PP789604	PP873244	PP873227	China	Chen et al. 2024
110.	*E. quadratum*	HFJAU2612	PP796419	PP789605	–	PP873228	China	Chen et al. 2024
111.	*E. quadratum*	HFJAU4223	PP796428	PP789611	PP873249	PP873233	China	Chen et al. 2024
112.	*E. quadratum*	HFJAU4265	PP796429	PP789612	PP873250	PP873234	China	Chen et al. 2024
113.	*E. quadratum*	HFJAU5173	PP796437	PP789620	PP873256	PP873240	China	Chen et al. 2024
114.	*E. quadratum*	HFJAU5179	PP796438	PP789621	PP873257	PP873241	China	Chen et al. 2024
115.	*E. quadratum*	iNAT:16890676	ON366783	–	–	–	USA	Unpublished
116.	*E. quadratum*	LE253781	–	MH190180	MH190118	–	Russia	Karstedt et al. 2019
117.	*E. quadratum*	LE254355	KC898452	KC898504	–	–	Russia	Morozova et al. 2014
118.	*E. quadratum*	PAN241	MZ611690	–	–	–	Panama	Reschke et al. 2022b
119.	*E. quadratum*	S.D. Russell ONT iNaturalist 136495142	OP749675	–	–	–	USA	Unpublished
120.	*E. quadratum*	WU21098	–	MW624828	–	–	France	Karstedt et al. 2024
121.	*E. rufomarginatum*	HFJAU1933, holotype	PP796415	PP789601	–	–	China	Chen et al. 2024
122.	*E. rufomarginatum*	HFJAU4070	PP883966	PP789608	–	–	China	Chen et al. 2024
**123.**	***E. rufosquamulosum***	**HKAS 69226**	**PX269824**	**PX269831**	–	–	**China**	**in this study**
**124.**	***E. rufosquamulosum***	**HKAS 130186, holotype**	**PX269818**	**PX269828**	**PX255543**	**PX255532**	**China**	**in this study**
**125.**	***E. rufosquamulosum***	**HKAS 136639**	**PX269822**	**PX269833**	**PX255547**	**PX255536**	**China**	**in this study**
**126.**	***E. rufosquamulosum***	**HKAS 143019**	**PX269823**	**PX269832**	**PX255548**	**PX255537**	**China**	**in this study**
**127.**	***E. rufosquamulosum***	**HKAS 150124**	**PX269821**	**PX269830**	**PX255546**	**PX255535**	**China**	**in this study**
**128.**	***E. rufosquamulosum***	**HKAS 150125**	**PX269820**	**PX269827**	**PX255545**	**PX255534**	**China**	**in this study**
**129.**	***E. rufosquamulosum***	**HKAS 150126**	**PX269819**	**PX269829**	**PX255544**	**PX255533**	**China**	**in this study**
130.	*E. semilanceatum*	NS2283	MN069544	–	–	–	Cameroon	Largent et al. 2020
131.	*E. sericeum*	KaiR237	OL338118	OL338542	OL405220	–	Germany	Reschke et al. 2022b
132.	*E. sericeum*	VHAs03/02	DQ367430	DQ367423	DQ367435	DQ367428		Hofstetter et al. 2014
133.	*E. smurfetti*	FK1709	–	MW624831	–	–	Brazil	Karstedt et al. 2024
134.	*E. smurfetti*	FK1717	–	MW624832	–	–	Brazil	Karstedt et al. 2024
135.	*E. smurfetti*	FK1741, holotype	–	MW624829	MW624751	MW624854	Brazil	Karstedt et al. 2024
136.	*E.* sp.	DLL9679	OR083035	KR233854	KR233915	–	Australia	Unpublished
137.	*E.* sp.	DLL9823	–	KR233861	KR233920	–	Australia	Unpublished
138.	*E. subcycneum*	HFJAU3124, holotype	PP796420	–	PP873245	PP873229	China	Chen et al. 2024
139.	*E. subcycneum*	HFJAU4738	PP796430	PP789613	–	–	China	Chen et al. 2024
140.	*E. submurrayi*	HFJAU1050	MN622719	–	–	–	China	Chen et al. 2024
141.	*E. submurrayi*	HFJAU3587, holotype	PP796423	PP789606	–	PP873230	China	Chen et al. 2024
142.	*E. tenue*	FK1922	–	MH190176	MH190115	–	Brazil	Karstedt et al. 2019
143.	*E. tomentosum*	HFJAU5159, holotype	PP796434	PP789617	PP873253	PP873237	China	Chen et al. 2024
144.	*E. tomentosum*	HFJAU5160	PP796435	PP789618	PP873254	PP873238	China	Chen et al. 2024
145.	*E. vinososquamulosum*	FK1745, holotype	–	MW624833	–	–	Brazil	Karstedt et al. 2024
146.	*E. virescens*	DLL9972	–	KR869937	KR869957	MG702628	Australia	Karstedt et al. 2019; Largent et al. 2016
147.	*E. virescens*	MCA2479	–	GU384622	GU384640	MG702629	Australia	Karstedt et al. 2019
148.	*E. virescens*	MEL:2379813	MF977981	–	–	–		Unpublished
149.	*E. voltavelhense*	FK1694, holotype	–	MW624834	MW624753	MW624856	Brazil	Karstedt et al. 2024
150.	*E. voltavelhense*	FK2118	–	MW624835	MW624754	–	Brazil	Karstedt et al. 2024
151.	*Inocephalus “argenteus*”	MCA1475	–	GU384619	GU384636	–		Baroni et al. 2011
152.	*I. squamulosus*	MCA1867	–	GU384621	GU384638	–		Baroni et al. 2011
153.	*Rhodophyllus lactifluus* (GB as *I. lactifluus*)	8753 TJB	–	MH190196	MH190129	MH190165	USA	Karstedt et al. 2019
154.	*R. lactifluus*	TB7962	–	AF261304	–	–		Moncalvo et al. 2002

### ﻿Taxonomy

#### ﻿ Entoloma acutiflavum

Taxon classificationFungiAgaricalesEntolomataceae

Z.W. Liu, Y.Y. Cui & Zhu L. Yang sp. nov.

64CFC14E-816F-550E-9268-90A348CF8924

860479

Figs 2, 3, 4

##### Diagnosis.

Pileus greyish yellow, with acuminate papilla at center. Basidiospores cuboid. Lamellar edge heterogeneous. Cheilocystidia elongated clavate, subcylindrical elongated clavate, or subcylindrical. Pileipellis with intracellular and incrusted pigment. Clamp connections present.

**Figure 2.**
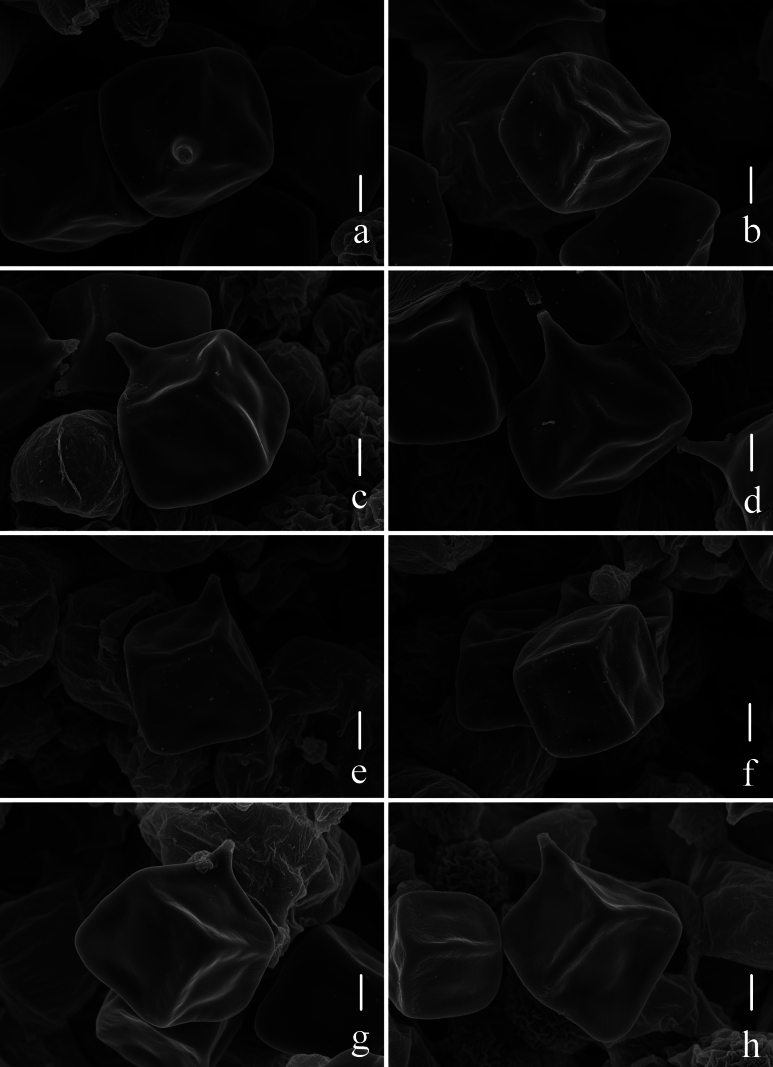
Basidiospores of four new species of Entoloma subg. Cubospora under SEM. a, b. *E. acutiflavum* HKAS 150128, holotype; c, d. *E. bichromum* HKAS 150132, holotype; e, f. *E. guttuliferum* HKAS 150129, holotype; g, h. *E. rufosquamulosum* HKAS 130186, holotype. Scale bars: 2 μm.

##### Holotype.

China • Yunnan Province: Baoshan, Longyang District, 25.2996°N, 98.7855°E, elev. 2200 m, in the litter layer of mixed broadleaf-conifer forest, 7 August 2022, Jin Li 085 (HKAS 150128).

**Figure 3.**
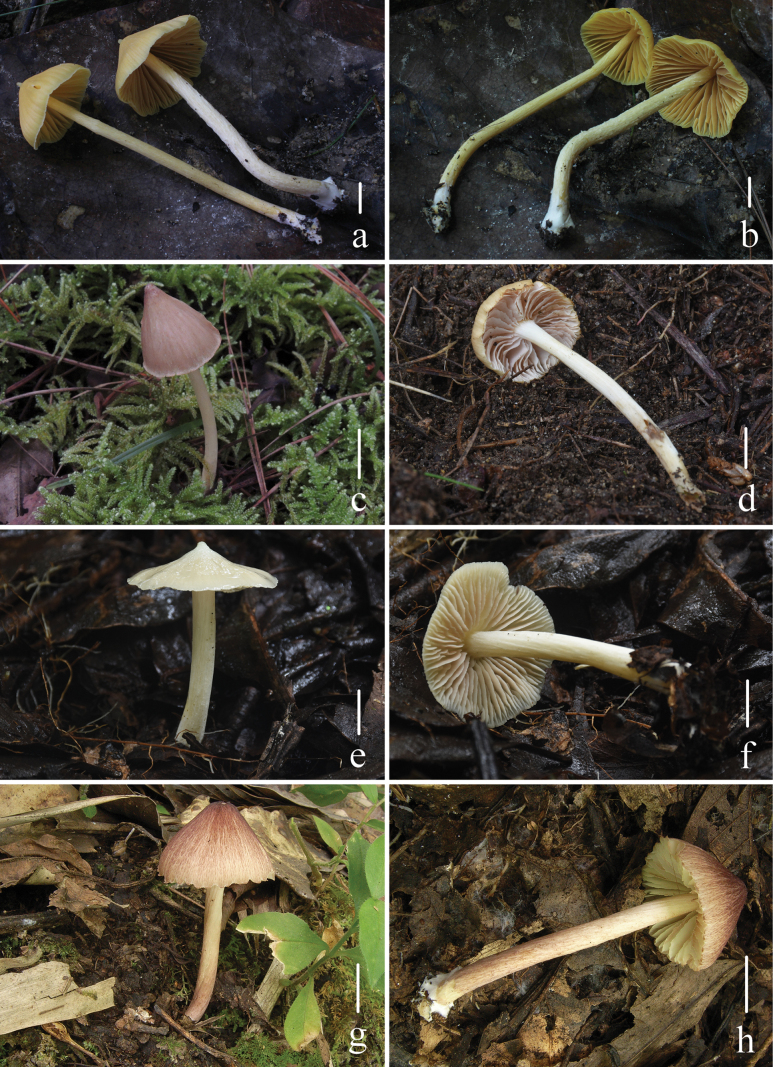
Basidiomata of four new species of Entoloma subg. Cubospora. a, b. *E. acutiflavum* HKAS 150128, holotype; c, d. *E. bichromum* (c) HKAS 150131, (d) HKAS 150132, holotype; e, f. *E. guttuliferum* HKAS 150129, holotype; g, h. *E. rufosquamulosum* HKAS 130186, holotype. Scale bars: 10 mm.

##### Etymology.

Refers to the yellowish pileus with acuminate papilla at center.

**Figure 4.**
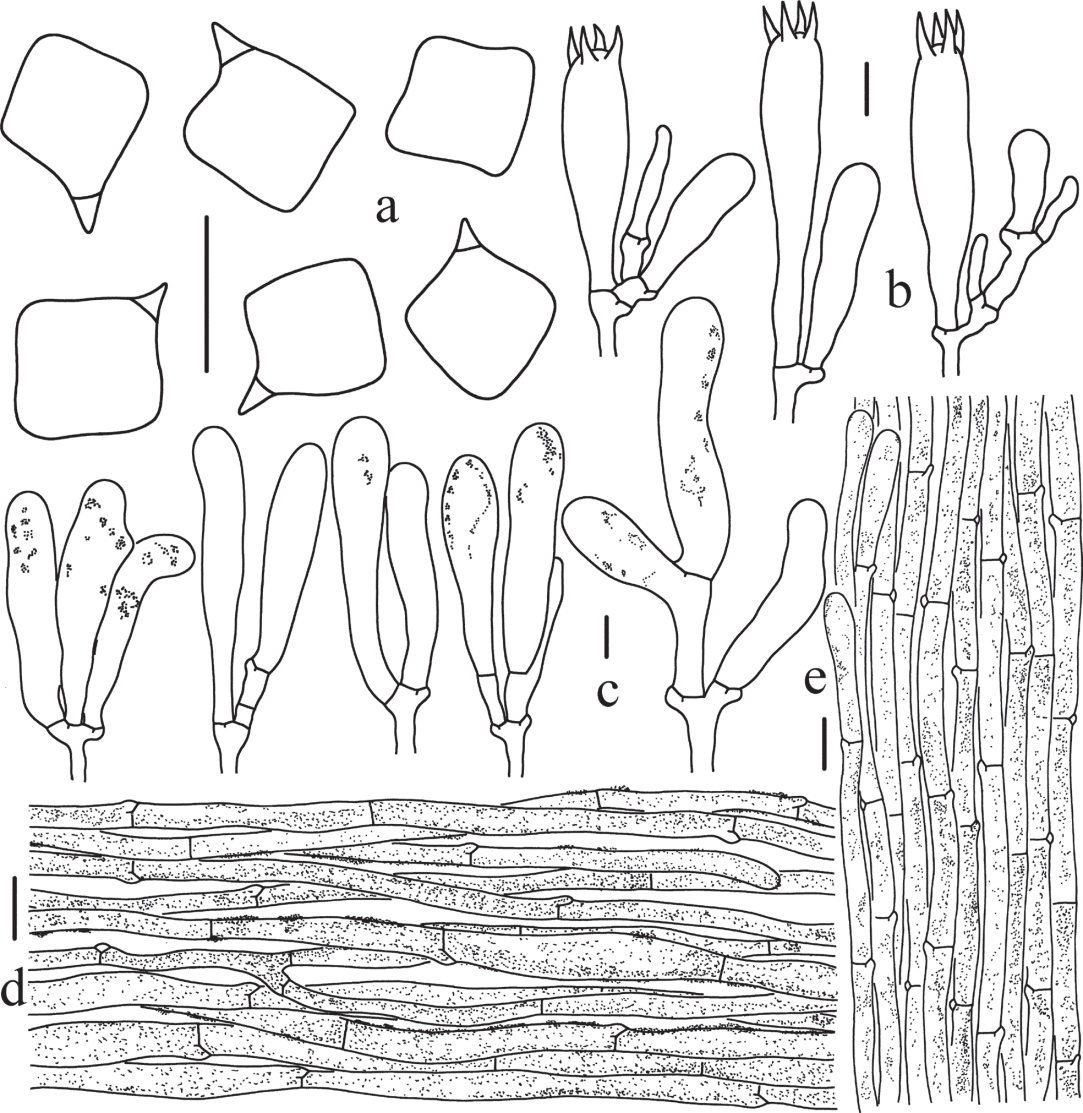
Microscopic features of *Entoloma acutiflavum* (HKAS 150128, holotype). a. Basidiospores; b. Basidia; c. Cheilocystidia; d. Pileipellis; e. Stipitipellis. Scale bars: 10 μm (a–c); 20 μm (d, e).

##### Description.

***Basidioma*** small to medium-sized. ***Pileus*** 28–42 mm in diam., conical, with acuminate papilla at center; greyish beige (4C2), light yellow (3A4) to greyish yellow (3C3, 4C5), margin concolorous or paler to yellowish white (2A2); striate sometimes shallowly sulcate, beige (4C3) or concolorous with pileus, towards the center up to 1/3 to 4/5 diam.; surface dry, smooth, occasionally hygrophanous, margin undulate; context thin. ***Lamellae*** sinuate, slightly crowded, 2–3 mm wide, with 1–3 tiers of lamellulae, narrowly ventricose, blond (4C4) or brownish orange (5C4), edge concolorous. ***Stipe*** 46–115 × 4–6 mm, central, cylindrical, usually tapering upwards, hollow; apex yellowish white (1A2) or greyish yellow (2C4, 3C3), sometimes downwards darker to beige (4C3); slightly with longitudinal or oblique striae, with white fibrils or smooth, base slightly swollen and with white mycelium. ***Odor*** and ***taste*** not observed.

***Basidiospores*** [69/2/2] (9.3)9.6–***10.9***–12.3(12.9) × (8.9)9.2–***10.4***–12.1(12.7) μm [Q = 1.00–1.12(1.20), **Q** = 1.05 ± 0.04], cuboid, most with 4 angles in side-view, rarely with 5 angles, lacking elongated angles. ***Basidia*** 45–79 × 11–22 μm, clavate, 4-spored, colorless. ***Lamellar edge*** heterogeneous. Cheilocystidia 28–89 × 7–18 μm, elongated clavate or subcylindrical, occasionally ventricose, hyaline or with pale yellowish green droplet-like content, thin-walled. Pleurocystidia absent. ***Lamellar trama*** regular, made up of cylindrical hyphae 3–12 μm diam., smooth, colorless. ***Pileipellis*** a cutis composed of cylindrical and fusiform hyphae, 2–16 μm wide, thin-walled, with yellowish brown intracellular pigment, also sometimes incrusting. ***Stipitipellis*** composed of longitudinally arranged, cylindrical hyphae 3–16 μm wide; terminal cells clavate to elongated clavate, 37–76 × 7–12 μm; both hyphae and terminal cells in stipitipellis with colorless intracellular content. ***Clamp connections*** present in all tissue. Refractive hyphae sometimes occur in lamellar trama.

##### Habitat.

Solitary or scattered in the litter layer of mixed broadleaf-conifer forests.

##### Known distribution.

Yunnan Province, China.

##### Additional material examined.

China • Yunnan Province: Baoshan, Longyang District, 25.2996°N, 98.7855°E, elev. 2200 m, in the litter layer of mixed broadleaf-conifer forest, 7 August 2022, Xue-Ping Fan 502 (HKAS 150127).

##### Notes.

*Entoloma acutiflavum* is similar to *E. submurrayi* and *E. murrayi* morphologically; meanwhile, *E. acutiflavum* and *E. submurrayi* form a close and stable clade in the phylogenetic tree. Compared to *E. submurrayi* reported from subtropical regions of China, *E. acutiflavum* differs in its dry pileus with brownish and orangish tinges, white fibrils on the stipe, heterogeneous lamellar edge, and encrusted pigment on the pileipellis (Chen et al. 2024). *Entoloma murrayi*, a widespread species, can be distinguished by its absence of clamp connections and sterile lamellar edge (Selby et al. 1859; Horak 1976; He 2012). In addition, *E. neotropicale* and *E. flavescens* have yellow basidiomata that are similar to those of *E. acutiflavum*. However, *E. neotropicale* has an appressed-fibrillose pileus with a more obtuse papilla and indistinct striations on the pileus margin (Karstedt et al. 2024). *Entoloma flavescens* possesses a shiny pileus partially covered with whitish silky scales, shorter cheilocystidia, and an almost hyaline pileipellis (Sato et al. 2025).

#### ﻿ Entoloma bichromum

Taxon classificationFungiAgaricalesEntolomataceae

Z.W. Liu, Y.Y. Cui & Zhu L. Yang sp. nov.

AC80EC4F-B667-563D-ACC5-029556B0C6E2

860480

Figs 2, 3, 5

##### Diagnosis.

Pileus with papilla at center and always dentate at margin. Lamellae edge serrate. Basidiospores cuboid. Pleurocystidia clavate, elongated clavate to cylindrical, always flexuous or furcate, sometimes ventricose. Clamp connections present.

**Figure 5.**
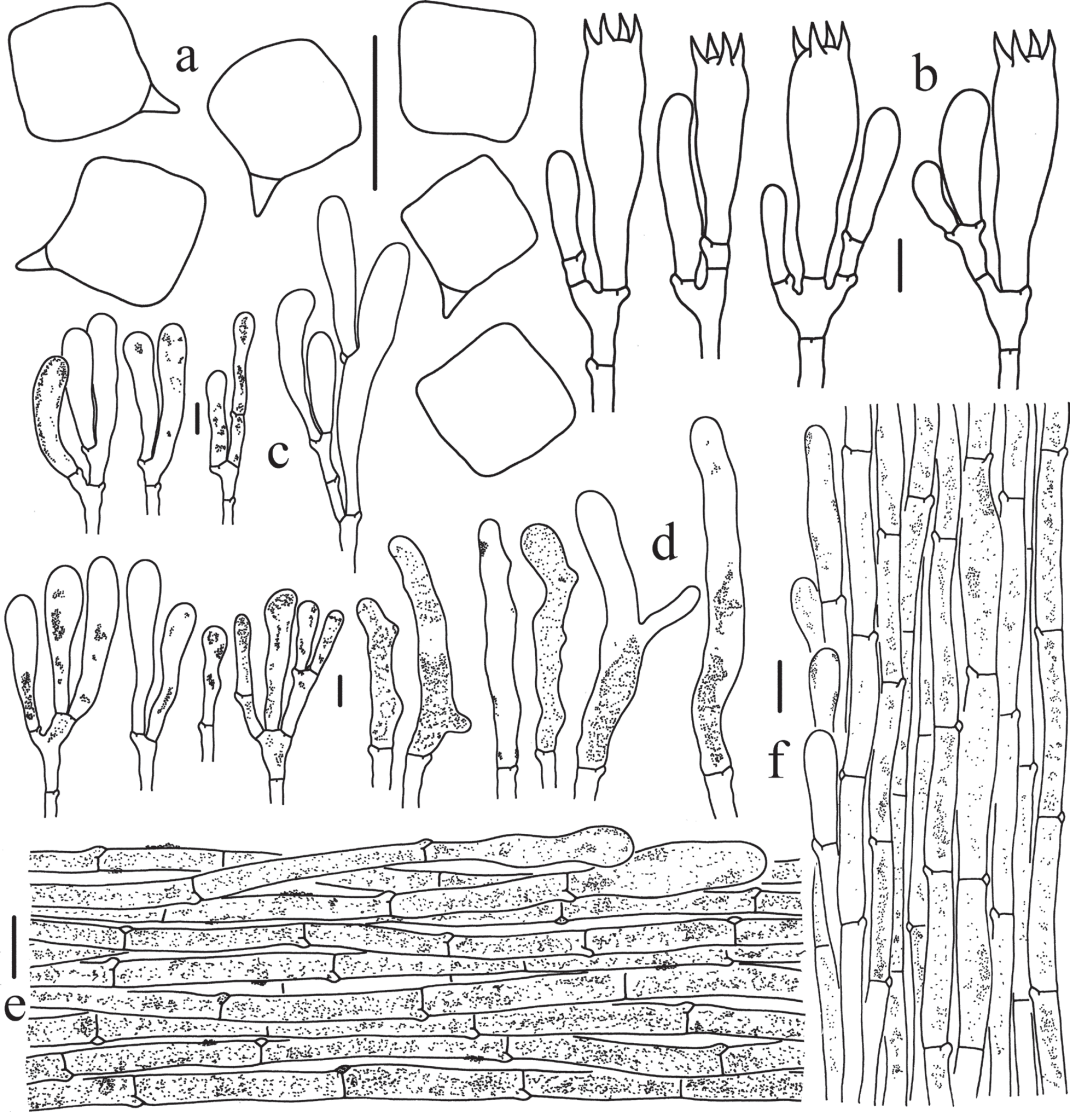
Microscopic features of *Entoloma bichromum* (HKAS 150132, holotype). a. Basidiospores; b. Basidia; c. Cheilocystidia; d. Pleurocystidia; e. Pileipellis; f. Stipitipellis. Scale bars: 10 μm (a–d); 20 μm (e, f).

##### Holotype.

China • Yunnan Province: Tengchong, Diantan Town, 25.2936°N, 98.4506°E, elev. 2200 m, on the soil in a mixed broadleaf-conifer forest, 27 July 2022, Peng-Cheng Yuan 892 (HKAS 150132).

##### Etymology.

Refers to the pileus exhibiting two color forms: reddish grey or orange white.

##### Description.

***Basidioma*** small. ***Pileus*** 16–26 mm in diam., conical to hemispherical, with a papilla at center, sometimes depressed around the papilla; greyish red (7B3, 11C4) or reddish brown (8E6) at center, disc becoming paler to reddish grey (10B2), light orange (6A4), or orange white (6A2); striations greyish orange (6B3) or greyish red (10D4), very near the center; surface dry, smooth, not hygrophanous, margin dentate. ***Lamellae*** sinuate to subdecurrent, slightly crowded, 1–4 mm wide, with 1–3 tiers of lamellulae, ventricose, pinkish white (10A2) to reddish grey (10B2), edge serrate and concolorous. ***Stipe*** 57–91 × 3–4 mm, central, cylindrical, hollow; apex to middle greyish yellow (4B3) or orange grey (6B2), base slightly darker to reddish grey (8B2) or greyish brown (5D3); smooth, with longitudinal striae, base slightly swollen and with white tomentum. ***Odor*** and ***taste*** not observed.

***Basidiospores*** [97/3/3] 8.8–***9.9***–10.9 × (8.2)8.6–***9.4***–10.2(10.8) μm [Q = 1.00–1.12, **Q** = 1.05 ± 0.03], cuboid, most with 4 angles in side-view, rarely with 5 angles, lacking elongated angles. ***Basidia*** 35–60 × 9–15 μm, clavate, 4-spored, colorless. ***Lamellar edge*** heterogeneous. Cheilocystidia 27–90 × 6–13 μm, clavate to elongated clavate, with pale yellowish green droplet-like content or hyaline, thin-walled. Pleurocystidia 27–116 × 6–13 μm, clavate, elongated clavate to cylindrical, always flexuous or furcate, sometimes ventricose, with pale yellowish green droplet-like content, sometimes hyaline, thin-walled. ***Lamellar trama*** regular, made up of cylindrical hyphae 3–17 μm diam., smooth, thin-walled, occasionally with droplet-like content. ***Pileipellis*** a cutis composed of cylindrical hyphae 2–15 μm wide, thin-walled; terminal cells clavate, 24–79 × 7–15 μm; both hyphae and terminal cells in pileipellis with colorless intracellular content, also occasionally incrusting. ***Stipitipellis*** composed of longitudinally arranged, cylindrical, and fusiform hyphae 4–14 μm wide; terminal cells clavate to elongated cylindrical, 20–74 × 5–12 μm; both hyphae and terminal cells in stipitipellis with colorless intracellular content. ***Clamp connections*** present in all tissue. Refractive hyphae sparsely present in lamellar trama.

##### Habitat.

Solitary in the litter layer or soil of mixed broadleaf-conifer or broadleaf forests.

##### Known distribution.

Yunnan Province, China.

##### Additional materials examined.

China • Yunnan Province: Baoshan, Longling County, 24.7793°N, 98.7926°E, elev. 1900–2000 m, in the litter layer of a mixed broadleaf-conifer forest with moss, 29 August 2022, Jin-Yan Tang 522 (HKAS 150131).

##### Notes.

*Entoloma bichromum* has a serrate lamellar edge, a grayish-red or orange-white pileus, and a concolorous or paler stipe. *Entoloma carneum* is phylogenetically closely related and morphologically similar to *E. bichromum* but can be distinguished by its lack of clamp connections and smaller spores (8–9 μm) (Bi et al. 1986). Moreover, *E. pallidoflavum*, *E. phlebophyllum*, *E. plicatum*, and *E. tomentosum* are close to the new species in the phylogenetic analysis but can be distinguished from *E. bichromum* based on the color of the basidiomata and the presence of squamules on the pilei (Horak 1976; Largent et al. 2013; Chen et al. 2024). *Entoloma laccarioides* is similar to *E. bichromum* in the color of the basidiomata, but the former has a subinfundibuliform pileus at maturity and fusoid to utriform pleurocystidia, whereas *E. bichromum* has a pileus always with a central papilla and clavate, elongated clavate to cylindrical, and narrower pleurocystidia (He et al. 2015a). In addition, *Entoloma peristerinum* resembles *E. bichromum* in pileus characters, but the former differs by its fibrillose stipe and the absence of pleurocystidia (Morozova and Pham 2023).

#### ﻿ Entoloma guttuliferum

Taxon classificationFungiAgaricalesEntolomataceae

Z.W. Liu, Y.Y. Cui & Zhu L. Yang sp. nov.

F9DAFB0B-51F1-5775-9979-3487AE3BCCFA

860481

Figs 2, 3, 6

##### Diagnosis.

Pileus yellowish white to white with a papilla at center. Basidiospores cuboid. Pleurocystidia clavate, occasionally septate, branched, or apically protruding. Pileipellis and stipitipellis with clavate or elongated clavate terminal cells. Cystidia and hyphae of lamellar trama, pileipellis, and stipitipellis with colorless intracellular content.

**Figure 6.**
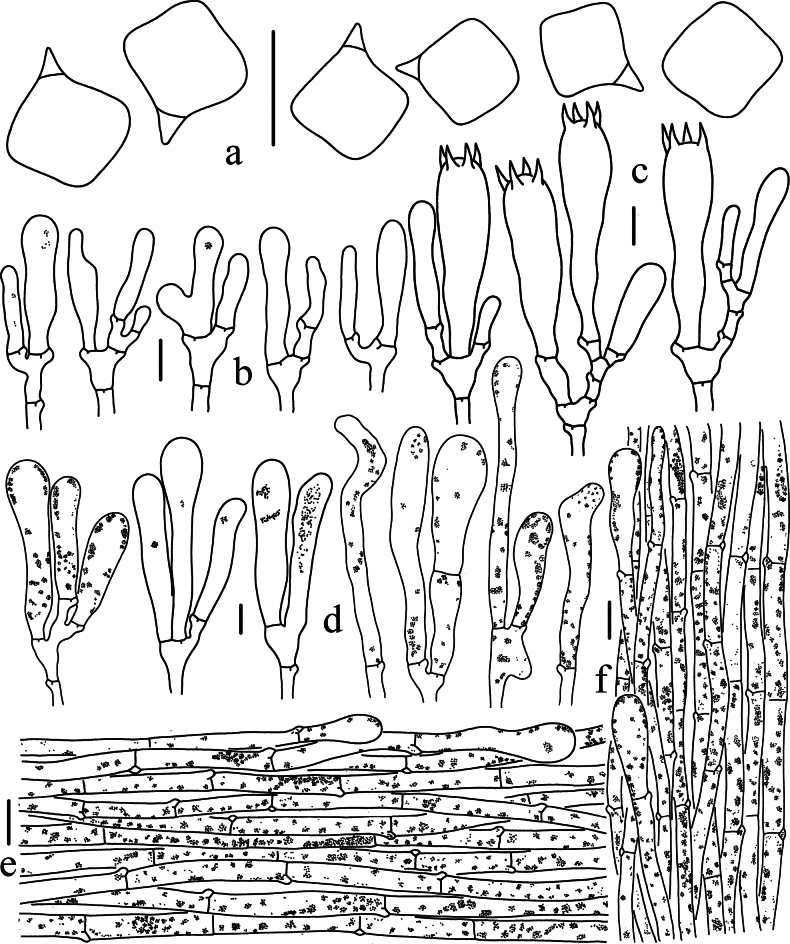
Microscopic features of *Entoloma guttuliferum* (HKAS 150129, holotype). a. Basidiospores; b. Pleurocystidia; c. Basidia; d. Cheilocystidia; e. Pileipellis; f. Stipitipellis. Scale bars: 10 μm (a–d); 20 μm (e, f).

##### Holotype.

China • Yunnan Province: Tengchong, Mingguang Town, 25.7456°N, 98.5594°E, elev. 2037 m, in the litter layer of broadleaf forest, 28 July 2022, Peng-Cheng Yuan 921 (HKAS 150129).

##### Etymology.

Refers to the droplet-like content distributed in multiple structures, including cystidia, hyphae of the lamellar trama, pileipellis, and stipitipellis.

##### Description.

***Basidioma*** small. ***Pileus*** 16–33 mm in diam., conical, convex to plano-convex, with a papilla at center; brownish (6D6–6D7), yellowish white (3A2) to white (1A1); striations indistinct, pale grey (1B1) to concolorous with pileus, towards the center up to 1/3–4/5 diam.; surface slightly lubricous, smooth, sometimes with tiny white tomentum at margin when young, not hygrophanous, margin undulate; context thin, white (1A1). ***Lamellae*** adnexed to sinuate, slightly crowded, 1–4 mm wide, with 3–5 tiers of lamellulae, narrowly ventricose, pinkish white (11A2), edge concolorous and sometimes serrated. ***Stipe*** 20–60 × 3–5 mm, central, cylindrical, hollow; greyish yellow (3C3) to olive (3D3) or medium grey (1D1, 1E1); with longitudinal or oblique striae, apex to middle with white tomentum or squamae, base slightly swollen and with white tomentum. ***Odor*** and ***taste*** not observed.

***Basidiospores*** [95/3/3] (8.5)9.2–***10.1***–11.0(12.0) × (8.0)8.6–***9.6***–10.4(10.8) μm [Q = 1.00–1.13(1.24), **Q** = 1.07 ± 0.05], cuboid, with 4 angles in side-view, lacking elongated angles. ***Basidia*** 48–70 × 9–17 μm, clavate, 4-spored, colorless. ***Lamellar edge*** heterogeneous. Cheilocystidia 26–189 × 7–15 μm, elongated clavate, sometimes flexuous, hyaline or with pale yellowish green droplet-like content, thin-walled. Pleurocystidia 18–56 × 3–10 μm, clavate, occasionally septate, branched, or apically protruding, hyaline, occasionally with droplet-like content, thin-walled. ***Lamellar trama*** regular, made up of cylindrical hyphae 5–18 μm diam., smooth, thick-walled, with droplet-like content. ***Pileipellis*** a cutis composed of cylindrical and fusiform hyphae 3–11 μm wide, thin-walled; terminal cells clavate, 20–88 × 4–15 μm; both hyphae and terminal cells in pileipellis with colorless droplet-like intracellular content. ***Stipitipellis*** composed of longitudinally arranged, cylindrical hyphae 3–13 μm wide; terminal cells clavate to elongated clavate, 24–71 × 4–17 μm; both hyphae and terminal cells in stipitipellis with colorless droplet-like intracellular content. ***Clamp connections*** present in all tissue. Refractive hyphae not observed.

##### Habitat.

Solitary in the litter layer of broadleaf forests, sometimes with moss.

##### Known distribution.

Yunnan Province, China.

##### Additional materials examined.

China • Yunnan Province: Tengchong, Mingguang Town, 25.7456°N, 98.5594°E, elev. 2037 m, in the litter layer of broadleaf forest, 28 July 2022, Xue-Ping Fan 350 (HKAS 150130); • Honghe Hani and Yi Autonomous Prefecture, Hekou Yao Autonomous County, elev. 2000 m, in the litter layer of broadleaf forest dominated by Fagaceae, 22 August 2019, Yun-Jiao Lüli 532532MF0183 (HKAS 107829).

##### Notes.

*Entoloma guttuliferum* is characterized by a yellowish-white to white pileus bearing a papilla and exhibiting indistinct striations, cuboid spores, and cystidia and hyphae of the lamellar trama, pileipellis, and stipitipellis with contents. *Entoloma guttuliferum* clusters with five species (*E. lacticolor*, *E. murrayi*, *E. submurrayi*, *E. acutiflavum*, and *E. quadratum*) in the phylogenetic tree. Among them, *E. lacticolor* is also similar to *E. guttuliferum* morphologically, but the former lacks pleurocystidia and has a sterile lamellar edge. Except for *E. lacticolor*, the other four species can be easily distinguished by the color of their basidiomata (Selby et al. 1859; Horak 1976; He 2012; Chen et al. 2024). *Entoloma albogracile* and *E. dennisii* share similarities with *E. guttuliferum* in pileus color and shape. However, *E. albogracile* differs in having pink to porphyry-pink lamellae, pigmentless cheilocystidia and pileipellis, and the absence of clamp connections. *Entoloma dennisii* has smaller spores (5.5–7 μm) and pigmentless cheilocystidia (Horak 1976). In addition, *E. album* and *E. caribaeum* also resemble *E. guttuliferum*, although the color of their pilei tends to be white. Compared to the new species, they lack pleurocystidia. Furthermore, the hyphae in the pileipellis of *E. album* lack contents. *Entoloma caribaeum* has larger spores with elongated angles (15–18 × 15–20 μm) and 2-spored basidia (Karstedt et al. 2024; Sato et al. 2025).

#### ﻿ Entoloma rufosquamulosum

Taxon classificationFungiAgaricalesEntolomataceae

Z.W. Liu, Y.Y. Cui & Zhu L. Yang sp. nov.

04348023-D6A3-5512-AD47-2D3144F91A73

860482

Figs 2, 3, 7

##### Diagnosis.

Pileus reddish with densely reddish brown or greyish violet squamae at center, becoming sparse towards margin. Basidiospores cuboid. Upper hyphae and terminal cells in pileipellis with reddish brown intracellular pigment.

**Figure 7.**
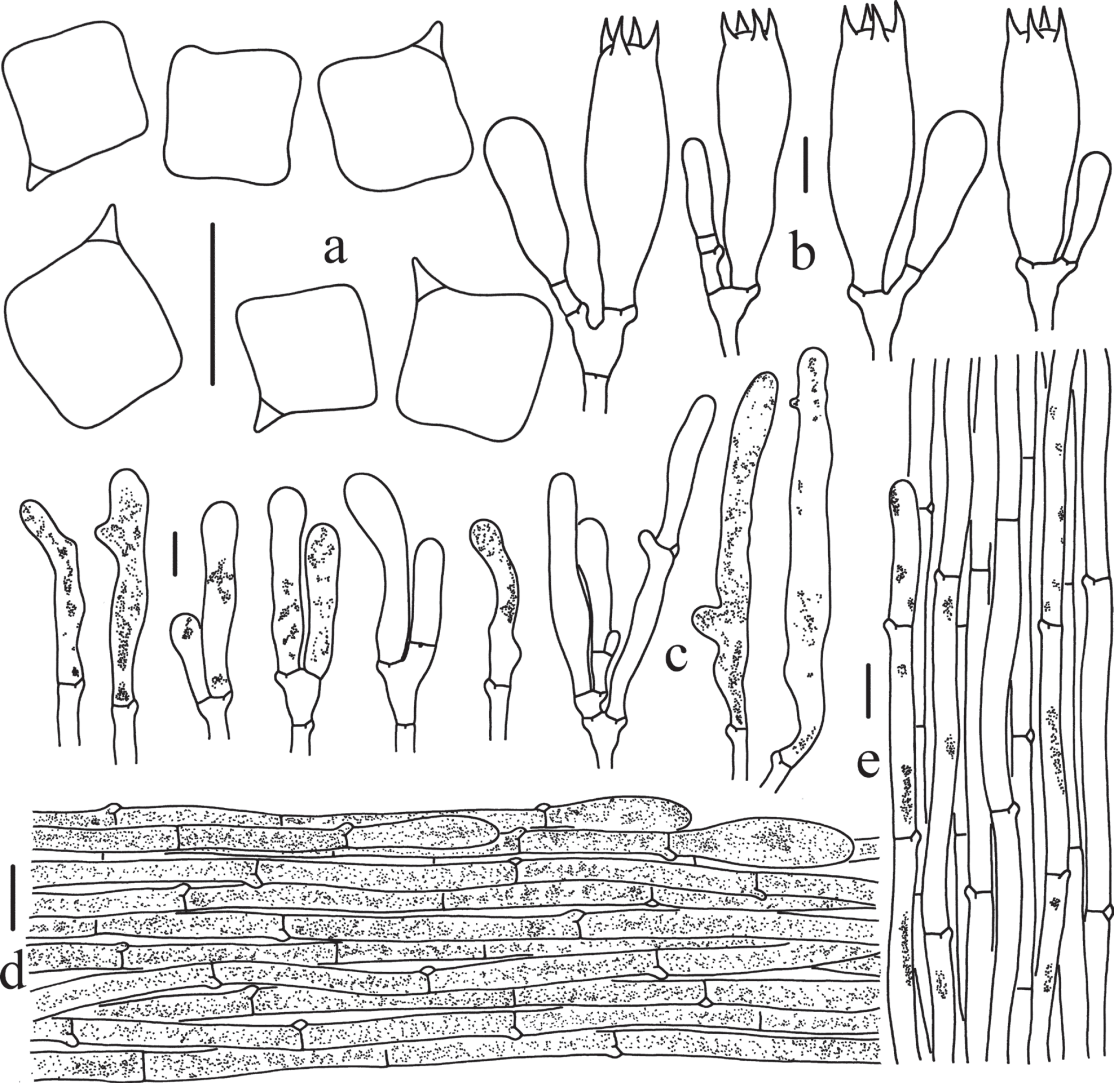
Microscopic features of *Entoloma rufosquamulosum* (HKAS 130186, holotype). a. Basidiospores; b. Basidia; c. Cheilocystidia; d. Pileipellis; e. Stipitipellis. Scale bars: 10 μm (a–c); 20 μm (d, e).

##### Holotype.

China • Yunnan Province: Nujiang Lisu Autonomous Prefecture, Fugong County, elev. 1750 m, in the litter layer of evergreen broad-leaved forest, 14 August 2023, Zhu L. Yang 7052 (HKAS 130186).

##### Etymology.

Refers to the pileus with densely reddish squamae.

##### Description.

***Basidioma*** small. ***Pileus*** 10–30 mm in diam., broadly conical to campanulate; violet brown (10E7) when young, becoming dull lilac (16C3), greyish magenta (13E4), or reddish brown (8E6) at center with age, disc paler to greyish lilac (16C2), greyish brown (8D3), reddish brown (8D4), or greyish ruby (12C3); striations none in most of specimens, occasionally present but indistinct, towards the center up to 1/3 to 3/4 diam.; surface dry, with densely reddish brown (8E7), greyish ruby (12E5) or greyish violet (18E5) squamae at center, becoming radial and gradually sparse towards margin, not hygrophanous, margin undulate, occasionally rimose and uplifted. ***Lamellae*** adnate to sinuate, crowded, 1–3 mm wide, with 1–3 tiers of lamellulae, narrowly ventricose, white to yellowish white (2A2), with pinkish tinged at age, edge serrate and concolorous. ***Stipe*** 22–114 × 3–6 mm, central, cylindrical, hollow, usually tapering upwards; greyish orange (5B3), reddish white (9A2), or greyish brown (8D3), sometimes with reddish brown (8D4) bruises; nearly smooth, sometimes with tiny granular ​squamules, with longitudinal or oblique striae, base slightly swollen and with white tomentum. ***Odor*** and ***taste*** not observed.

***Basidiospores*** [110/4/4] (9.0)9.4–***10.3***–11.2(11.8) × (8.3)8.9–***9.7***–10.6(11.0) μm [Q = 1.00–1.14(1.18), **Q** = 1.06 ± 0.04], cuboid, most with 4 angles in side-view, rarely with 5 angles, lacking elongated angles. ***Basidia*** 37–59 × 12–18 μm, clavate, 4-spored, colorless. ***Lamellar edge*** heterogeneous. Cheilocystidia 21–98 × 6–13 μm, clavate to elongated clavate, sometimes flexuous or furcate, with pale yellowish green droplet-like content or hyaline, thin-walled. Pleurocystidia absent. ***Lamellar trama*** regular, made up of cylindrical hyphae 3–15 μm diam., smooth, thin-walled, sometimes with droplet-like content. ***Pileipellis*** a cutis composed of cylindrical hyphae 3–20 μm wide, thin-walled; terminal cells clavate or fusiform, 27–111 × 6–17 μm; both upper hyphae and terminal cells in pileipellis with reddish brown intracellular pigment, lower hyphae with colorless intracellular content. ***Stipitipellis*** composed of longitudinally arranged, cylindrical hyphae 3–17 μm wide; terminal cells sparse, elongated cylindrical, 44–68 × 9–13 μm; both hyphae and terminal cells in stipitipellis with colorless intracellular content. ***Clamp connections*** present in all tissue. Refractive hyphae occasionally occur in lamellar trama and pileipellis.

##### Habitat.

Solitary or scattered in the litter layer of evergreen broad-leaved forest, coniferous forest dominated by *Keteleeria fortunei*, or mixed broadleaf-conifer forests.

##### Known distribution.

Yunnan Province, China.

##### Additional materials examined.

China • Yunnan Province: Tengchong, Zhonghe Town, elev. 1970 m, in the litter layer in coniferous forest dominated by *Keteleeria fortunei*, 13 August 2010, Yan-Jia Hao 242 (HKAS 69226); • Mingguang Town, 25.7456°N, 98.5594°E, elev. 2037 m, in the litter layer of broadleaf forest, 28 July 2022, Peng-Cheng Yuan 917 (HKAS 143019); • Diantan Town, 25.2936°N, 98.4506°E, elev. 2170–2220 m, in the litter layer of broadleaf-conifer forest, 27 July 2022, Xue-Ping Fan 316 (HKAS 136639); • Baoshan, Longyang District, 25.2996°N, 98.7855°E, elev. 2200 m, in the litter layer of broadleaf-conifer forest, 7 August 2022, Xue-Ping Fan 540 (HKAS 150126); • Longling County, 24.7789°N, 98.7931°E, elev. 1950 m, in the litter layer of broadleaf-conifer forest, 29 August 2022, Xue-Ping Fan 769 (HKAS 150125); • Longling County, 24.9478°N, 98.6108°E, elev. 2050 m, in the litter layer of broadleaf-conifer forest, 1 September 2022, Xue-Ping Fan 931 (HKAS 150124).

##### Notes.

*Entoloma rufosquamulosum* is characterized by its pileus with reddish or violet squamules and cuboid spores. *E. rufosquamulosum* forms a stable clade with *E. bichromum*, *E. carneum*, *E. pallidoflavum*, *E. phlebophyllum*, *E. plicatum*, and *E. tomentosum* in the phylogenetic tree. Among them, *E. phlebophyllum* resembles *E. rufosquamulosum* when young, but the former has a pileus with a depressed center at maturity, a sterile lamellar edge, and a pileipellis with encrusted pigment (Chen et al. 2024). *Entoloma carneum* has been reported to have a pale carneous pileus with a red center and radiating fibers, but it differs greatly from *E. rufosquamulosum* in microstructure, including smaller spores, the presence of pleurocystidia, and the absence of clamp connections (Bi et al. 1986). *Entoloma bichromum*, *E. pallidoflavum*, *E. plicatum*, and *E. tomentosum* can be easily distinguished from *E. rufosquamulosum* by the distinctly different colors of the pileus and stipe (Horak 1976; Largent et al. 2013; Chen et al. 2024). In addition, three species from Brazil—*E. arenicola*, *E. vinososquamulosum*, and *E. voltavelhense*—are similar to *E. rufosquamulosum* macroscopically. However, they have smaller spores or straw-yellow intracellular pigment in the pileipellis (Karstedt et al. 2024).

### ﻿Key to species of Entoloma subg. Cubospora from China

**Table:** 

1	Pileus yellow, orange or brown	**2**
–	Pileus other colored	**12**
2	Pileus with obvious acute papilla at center	**3**
–	Pileus obtuse at the apex or depressed at center	**8**
3	Lamellae edge with red-brown underlined	***Entoloma rufomarginatum***
–	Lamellae edge without red-brown underlined	**4**
4	Hyphae of pileipellis hyaline	**5**
–	Hyphae of pileipellis with pigment	**6**
5	Clamps absent	***Entoloma murrayi***
–	Clamps present	***Entoloma flavescens***
6	Pileus rimose-fibrillose and orange	***Entoloma quadratum***
–	Pileus smooth and yellow	**7**
7	Lamellae edge heterogeneous	***Entoloma acutiflavum***
–	Lamellae edge sterile	***Entoloma submurrayi***
8	Pileus and stipe densely covered with scales	***Entoloma petchii***
–	Pileus and stipe glabrous, slightly fibrillose or partially pruinose	**9**
9	Spores less than 7.5 μm length	**10**
–	Spores more than 7.5 μm length	**11**
10	Cheilocystidia present	***Entoloma cuboidosporum***
–	Cheilocystidia absent	***Entoloma conspicuum***
11	Lamellae bright yellow, adnexed to sinuate	***Entoloma excavatum***
–	Lamellae white to pale salmon, adnate to almost free	***Entoloma luteum***
12	Pileus white to beige	**13**
–	Pileus other colored	**19**
13	Lamellae short decurrent; cheilocystidia absent	***Entoloma pseudogriseoalbum***
–	Lamellae not short decurrent; cheilocystidia present	**14**
14	Pleurocystidia present	**15**
–	Pleurocystidia absent	**16**
15	Pileus plano-convex, depressed at center with age	***Entoloma laccarioides***
–	Pileus conical to campanulate, with papilla at center	***Entoloma guttuliferum***
16	Spores with elongated angles	***Entoloma album***
–	Spores without elongated angles	**17**
17	Lamellae edge heterogeneous	***Entoloma pseudotomentosum***
–	Lamellae edge sterile	**18**
18	Pileus with obvious acute papilla at the center and more than 15 mm in diam.; hyphae of pileipellis without pigment	***Entoloma lacticolor***
–	Pileus without obvious acute papilla at the center and less than 15 mm in diam.; hyphae of pileipellis with pale yellow encrusting pigment	***Entoloma subcycneum***
19	Pileus red to purple	**20**
–	Pileus blue to green	**23**
20	Pileus smooth	***Entoloma bichromum***
–	Pileus squamulose or fibrillose	**21**
21	Clamps absent	***Entoloma carneum***
–	Clamps present	**22**
22	Pileus depressed at center; lamellae edge sterile	***Entoloma phlebophyllum***
–	Pileus not depressed at center; lamellae edge heterogeneous	***Entoloma rufosquamulosum***
23	Hyphae of pileipellis with bluish intracellular pigment	**24**
–	Hyphae of pileipellis with pale yellowish or stramineous intracellular pigment	**25**
24	Pileus non-striate and fibrillose; lamellae crowded; spores smaller (7–10.5 μm); cheilocystidia longer and narrower, cylindrical to clavate	***Entoloma altissimum***
–	Pileus slightly striate and glabrous to weakly furfuraceous; lamellae distant to subdistant; spores larger (8–12.5 μm); cheilocystidia shorter and wider, broadly clavate	***Entoloma subaltissimum***
25	Spores less than 8 μm length	***Entoloma mengsongense***
–	Spores more than 8 μm length	***Entoloma virescens***

## ﻿Discussion

Entoloma subg. Cubospora was established by Karstedt et al. (2019) to accommodate species characterized by cuboid basidiospores; mycenoid, collybioid, or tricholomatoid basidiomata; cylindrical-clavate or clavate cheilocystidia; and a trichodermal to cutis-like pileipellis. Later, Karstedt et al. (2024) expanded the subgenus with numerous new taxa from Brazil and proposed seven subclades based on LSU, mtSSU, *rpb2*, and *tef1* data, although two subclades were weakly supported. Chen et al. (2024) subsequently described seven additional species of E. subg. Cubospora from subtropical regions of China based on LSU, *rpb2*, and *tef1* analyses. Incorporating these taxa, the present study confirms the monophyly and morphological coherence of E. subg. Cubospora as defined by Karstedt et al. (2019). Our phylogenetic results also lend partial support to the subdivision proposed by Karstedt et al. (2024), though subclade /Murrayi exhibited weak support (BS/BPP = 37/0.535). Based on pileus coloration, the presence of cuboid basidiospores with non-elongated angles, and phylogenetic placement, the four newly described species fit within subclade /Murrayi. However, *E. acutiflavum*, *E. bichromum*, and *E. guttuliferum* possess smooth to nearly smooth pilei, differing from the appressed-fibrillose pileus typical of that group (Karstedt et al. 2024). Further taxonomic resolution and subclade delimitation will require additional sampling and data.

Many specimens exhibiting yellow, orange, or white basidiomata have been historically identified as *E. murrayi*, *E. quadratum*, or *E. album*. However, these names likely encompass multiple undescribed species. For instance, *E. submurrayi* (Chen et al. 2024) and *E. acutiflavum* (this study) were both collected from subtropical China, highlighting the region’s underexplored fungal diversity. Although the view that cuboid-spored species are primarily distributed in tropical to subtropical regions is widely accepted (Horak 1976, 1977; Romagnesi and Gilles 1979; He et al. 2015a; Reschke et al. 2022a; Chen et al. 2024), recent findings challenge this opinion. Sato et al. (2025) described temperate-zone taxa morphologically similar to *E. murrayi* and *E. quadratum*, supporting the view that cuboid-spored species also occur in temperate regions. This observation aligns with early records such as *E. luteum* from northwestern North America (Peck 1902), further emphasizing the ecological diversity of this distinctive lineage.

## Supplementary Material

XML Treatment for Entoloma acutiflavum

XML Treatment for Entoloma bichromum

XML Treatment for Entoloma guttuliferum

XML Treatment for Entoloma rufosquamulosum

## ﻿Additional information

### Use of AI

No use of AI was reported.

### Author ORCIDs

Ze-Wei Liu https://orcid.org/0000-0002-2700-0831

Jing Li https://orcid.org/0000-0002-0966-0166

Zhu L. Yang https://orcid.org/0000-0001-9745-8453

Yang-Yang Cui https://orcid.org/0000-0003-3990-8342

### Data availability

All of the data that support the findings of this study are available in the main text.

## References

[B1] Baroni TJ (1981) A revision of the genus *Rhodocybe* Maire (Agaricales) (Beihefte zur Nova Hedwigia, Vol. 67). Johann Christian Cramer Verlag, Stuttgart, 194 pp.

[B2] Baroni TJ Hofstetter V Largent DL Vilgalys R (2011) *Entocybe* is proposed as a new genus in the Entolomataceae (Agaricomycetes, Basidiomycota) based on morphological and molecular evidence. North American Fungi 6(12): 1–19. 10.2509/naf2011.006.012

[B3] Bi ZS Zheng GY Li TH (1986) Taxonomic studies on the genus *Entoloma* from Guangdong. Acta Mycologica Sinica 5: 161–169.

[B4] Chen ZH Zhang P (2019) Atlas of Macrofung in Hunan. Hunan Normal University Press, Changsha, 426 pp.

[B5] Chen LG Ding L Chen H Zeng H Zeng ZH Wang SN Yan JQ (2024) Seven new species of Entoloma subgenus Cubospora (Entolomataceae, Agaricales) from Subtropical Regions of China. Journal of Fungi 10: 594. 10.3390/jof10080594 PMC11355251 39194919

[B6] Clémençon H Emmett V Emmett EE (2004) Cytology and Plectology of the Hymenomycetes. Bibliotheca Mycologica, Vol. 199. Johann Christian Cramer Verlag, Stuttgart, 488 pp.

[B7] Co-David D Langeveld D Noordeloos ME (2009) Molecular phylogeny and spore evolution of Entolomataceae. Persoonia 23: 147176. 10.3767/003158509X480944 PMC2802732 20198166

[B8] Crous PW Osieck ER Jurjevi Ž Boers J Van Iperen AL Starink-Willemse M Dima B Balashov S Bulgakov TS Johnston PR Morozova OV Pinruan U Sommai S Alvarado P Decock CA Lebel T McMullan-Fisher S Moreno G Shivas RG Zhao L Abdollahzadeh J Abrinbana M Ageev DV Akhmetova G Alexandrova AV Altés A Amaral AGG Angelini C Antonín V Arenas F Asselman P Badali F Baghela A Bañares A Barreto RW Baseia IG Bellanger J-M Berraf-Tebbal A Biketova A Yu Bukharova NV Burgess TI Cabero J Câmara MPS Cano-Lira JF Ceryngier P Chávez R Cowan DA de Lima AF Oliveira RL Denman S Dang QN Dovana F Duarte IG Eichmeier A Erhard A Esteve-Raventós F Fellin A Ferisin G Ferreira RJ Ferrer A Finy P Gaya E Geering ADW Gil-Durán C Glässnerová K Glushakova AM Gramaje D Guard FE Guarnizo AL Haelewaters D Halling RE Hill R Hirooka Y Hubka V Iliushin VA Ivanova DD Ivanushkina NE Jangsantear P Justo A Kachalkin AV Kato S Khamsuntorn P Kirtsideli IY Knapp DG Kochkina GA Koukol O Kovács GM Kruse J Kumar TKA Kušan I Læssøe T Larsson E Lebeuf R Levicán G Loizides M Marinho P Luangsa-ard JJ Lukina EG Magaña-Dueñas V Maggs-Kölling G Malysheva EF Malysheva VF Martín B Martín MP Matočec N McTaggart AR Mehrabi-Koushki M Mešić A Miller AN Mironova P Moreau P-A Morte A Müller K Nagy LG Nanu S Navarro-Ródenas A Nel WJ Nguyen TH Nóbrega TF Noordeloos ME Olariaga I Overton BE Ozerskaya SM Palani P Pancorbo F Papp V Pawłowska J Pham TQ Phosri C Popov ES Portugal A Pošta A Reschke K Reul M Ricci GM Rodríguez A Romanowski J Ruchikachorn N Saar I Safi A Sakolrak B Salzmann F Sandoval-Denis M Sangwichein E Sanhueza L Sato T Sastoque A Senn-Irlet B Shibata A Siepe K Somrithipol S Spetik M Sridhar P Stchigel AM Stuskova K Suwannasai N Tan YP Thangavel R Tiago I Tiwari S Tkalčec Z Tomashevskaya MA Tonegawa C Tran HX Tran NT Trovão J Trubitsyn VE Van Wyk J Vieira WAS Vila J Visagie CM Vizzini A Volobuev SV Vu DT Wangsawat N Yaguchi T Ercole E Ferreira BW de Souza AP Vieira BS Groenewald JZ (2021) Fungal Planet description sheets: 1284–1382. Persoonia 47: 178–374. 10.3767/persoonia.2021.47.06 37693795 PMC10486635

[B9] Ediriweera AN Karunarathna SC Xu JC Hyde KD Mortimer PE (2017) *Entoloma mengsongense* sp. nov. (Entolomataceae, Agaricales), a remarkable blue mushroom from Yunnan Province, China. Turkish Journal of Botany 41: 505–515. 10.3906/bot-1611-13

[B10] Edler D Klein J Antonelli A Silvestro D (2021) raxmlGUI 2.0: A graphical interface and toolkit for phylogenetic analyses using RAxML. Methods in Ecology and Evolution 12(2): 373–377. 10.1111/2041-210X.13512

[B11] Feng WC Li XY Cui YY Cai Q (2025) Species diversity of Gymnopus section Levipedes in southwestern China, with a description of three new species. Journal of Fungi 11: 88. 10.3390/jof11020088 PMC11856841 39997382

[B12] Gardes M Bruns TD (1993) ITS primers with enhanced speciﬁcity for basidiomycetes—Application to the identiﬁcation of mycorrhizae and rusts. Molecular Ecology 2: 113–118. 10.1111/j.1365-294X.1993.tb00005.x 8180733

[B13] Gates GM Noordeloos ME (2007) Preliminary studies in the genus *Entoloma* in Tasmania– I. Persoonia 19: 157–226.

[B14] Gates GM Horton BM Noordeloos ME (2009) A new *Entoloma* (Basidiomycetes, Agaricales) from Tasmania. Mycotaxon 107: 175–179. 10.5248/107.175

[B15] Hall TA (1999) BioEdit: A user-friendly biological sequence alignment editor and analysis program for Windows 95/98/NT. Nucleic Acids Symposium Series 41: 95–98.

[B16] He XL (2012) Taxonomy of *Entoloma* in China and molecular phylogeny on Entolomataceae. PhD Thesis, South China Agricultural University, Guangdong, China.

[B17] He XL Li TH Jiang ZD Shen YH (2012) Four new species of *Entoloma* s.l. (Agaricales) from southern China. Mycological Progress 11: 915–925. 10.1007/s11557-012-0807-0

[B18] He XL Li TH Xi PG Jiang ZD Shen YH (2013) Phylogeny of Entoloma s.l. subgenus Pouzarella, with descriptions of five new species from China. Fungal Diversity 58: 227–243. 10.1007/s13225-012-0212-7

[B19] He XL Li TH Jiang ZD Shen YH (2015a) Two new cuboid-spored species of *Entoloma* s.l. (Agaricales, Entolomataceae) from southern China. Cryptogamie. Mycologie 36: 237–249.

[B20] He XL Ye XJ Li TH Peng WH Gan BC (2015b) New and noteworthy species of white *Entoloma* (Agaricales, Entolomataceae) in China. Phytotaxa 205(2): 99–110. 10.11646/phytotaxa.205.2.3

[B21] Hernández A (2017) Estudio taxonómico de algunos macromicetos del orden agaricales del Volcán Tacaná, Chiapas Lili Jazbeth Arias Hernández. Master’s Thesis, El Colegio de la Frontera Sur (ECOSUR), Chiapas, México.

[B22] Hofstetter V Redhead SA Kauff F Moncalvo J Matheny PB Vilgalys R (2014) Taxonomic revision and examination of ecological transitions of the Lyophyllaceae (Basidiomycota, Agaricales) based on a multigene phylogeny. Cryptogamie. Mycologie 35(4): 399–425. 10.7872/crym.v35.iss4.2014.399

[B23] Horak E (1976) On cuboid spored species of *Entoloma* (Agaricales). Sydowia 28: 171–236.

[B24] Horak E (1977) Additions to “On cuboid-spored species of *Entoloma*”. Sydowia 29: 289–299.

[B25] Horak E (1980) *Entoloma* (Agaricales) in Indomalaya and Australasia (Beihefte Nova Hedwigia Vol. 65). Johann Christian Cramer Verlag, Stuttgart, 352 pp.

[B26] Horak E (2005) Röhrlinge und Blätterpilze in Europa: Bestimmungsschlüssel für Polyporales (p.p.), Boletales, Agaricales, Russulales. Elsevier, Amsterdam. Spektrum Akademischer Verlag, Jena, 555 pp.

[B27] Horak E (2008) Agaricales of New Zealand 1: Pluteaceae – Entolomataceae. In The Fungi of New Zealand Vol. 5. Fungal Diversity Press, Hong Kong, 305 pp.

[B28] Jian SP (2020) The application of DNA barcoding technology in species identification of *Clitopilus*, *Clitocella* and *Clitopilopsis*. Master’s Thesis, Yunnan University, Yunnan, China.

[B29] Karstedt F Capelari M Baroni TJ Largent DL Bergemann SE (2019) Phylogenetic and morphological analyses of species of the Entolomataceae (Agaricales, Basidiomycota) with cuboid basidiospores. Phytotaxa 391(1): 001–027. 10.11646/phytotaxa.391.1.1

[B30] Karstedt F Bergemann SE Capelari M (2020) Five *Nolanea* spp. nov. from Brazil. Mycotaxon 135: 589–612. 10.5248/135.589

[B31] Karstedt F Bergemann SE Gates G Ratkowsky D Cunha KM Capelari M (2024) Species of *Entoloma* (Entolomataceae) with cuboidal basidiospores from Brazil. Phytotaxa 654(1): 001–076. 10.11646/phytotaxa.654.1.1

[B32] Katoh K Standley DM (2013) MAFFT multiple sequence alignment software version 7: Improvements in performance and usability. Molecular Biology and Evolution 30(4): 772–780. 10.1093/molbev/mst010 23329690 PMC3603318

[B33] Kim CS Jo JW Kwag Y Sung G Lee S Kim S Shin C Han S (2015) Mushroom flora of Ulleung-gun and a newly recorded *Bovista* species in the Republic of Korea. Mycobiology 43(3): 239–257. 10.5941/MYCO.2015.43.3.239 26539040 PMC4630430

[B34] Kluting KL Baroni TJ Bergemann SE (2014) Toward a stable classification of genera within the Entolomataceae: A phylogenetic re-evaluation of the *Rhodocybe-Clitopilus* Clade. Mycologia 106(6): 1127–1142. 10.3852/13-270 24987124

[B35] Kornerup A Wanscher JH (1978) Methuen handbook of colour. Eyre Methuen, London, 252 pp.

[B36] Largent DL (1977) The genus *Leptonia* on the Pacific Coast of the United States: Including a study of the North American types (Bibliotheca mycologica, Vol. 55). Johann Christian Cramer Verlag, Stuttgart, 286 pp.

[B37] Largent DL (1994) Entolomatoid fungi of the Western United States and Alaska. Mad River Press, Eureka, 495 pp.

[B38] Largent DL Bergemann SE Abell-Davis SE Kluting KL Cummings GA (2013) Three new *Inocephalus* species with cuboid basidiospores from New South Wales and Queensland, Australia. Mycotaxon 123: 301–319. 10.5248/123.301

[B39] Largent DL Kluting KL Anderson NM Bergemann SE (2016) New leptonioid species from New South Wales and northeastern Queensland, Australia. Mycotaxon 131: 153–176. 10.5248/131.153

[B40] Largent DL Henkel TW Siegel N Koch RA Séné O Hageman KM Aime MC (2020) New species of Entolomataceae from Cameroon. Fungal Systematics and Evolution 5: 151–167. 10.3114/fuse.2020.05.10 32467921 PMC7250018

[B41] Liu ZW Ge YP Zeng H Cheng XH Na Q (2022) Four new species of Mycena sect. Calodontes (Agaricales, Mycenaceae) from northeast China. MycoKeys 93: 23–56. 10.3897/mycokeys.93.86580 36761907 PMC9849094

[B42] Moncalvo JM Vilgalys R Redhead SA Johnson JE James TY Aime MC Hofstetter V Verduin S Larsen E Baroni TJ Thorn RG Jacobsson S Clémençon H Miller Jr OK (2002) One hundred and seventeen clades of euagarics. Molecular Phylogenetics and Evolution 23: 357–400. 10.1016/S1055-7903(02)00027-1 12099793

[B43] Morozova O Pham THG (2023) New Species of *Entoloma* subgenera *Cubospora* and *Leptonia* (Agaricales, Basidiomycota) from Central Vietnam. Journal of Fungi 9: 621. 10.3390/jof9060621 PMC10303506 37367557

[B44] Morozova O Noordeloos ME Vila J (2014) Entoloma subgenus Leptonia in boreal-temperate Eurasia: Towards a phylogenetic species concept. Persoonia 32: 141–169. 10.3767/003158514X681774 25264388 PMC4150075

[B45] Noordeloos ME (1988) Entolomataceae. In: Bas C Kuyper TW Noordeloos ME Vellinga EC (Eds) Flora agaricina neerlandica (Vol.1). Balkema, Rotterdam, 77–182.

[B46] Noordeloos ME (1992) *Entoloma* s.l. Fungi Europaei (Vol. 5). Candusso Edizioni, Origgio (VA), 760 pp.

[B47] Noordeloos ME (2004) *Entoloma* s.l. Fungi Europaei (Vol. 5a). Candusso Edizioni, Origgio (VA), 618 pp.

[B48] Noordeloos ME Gates GM (2009) Preliminary studies in the genus *Entoloma* of Tasmania II. Cryptogamie. Mycologie 30: 107–140.

[B49] Noordeloos ME Gates GM (2012) The Entolomataceae of Tasmania. Springer Science & Business Media, Berlin, 400 pp. 10.1007/978-94-007-4679-4

[B50] Noordeloos ME Hausknecht A (2007) The genus *Entoloma* (Basidiomcyetes, Agaricales) of the Mascarenes and Seychelles. Fungal Diversity 27: 111–144.

[B51] Noordeloos ME Weholt Ø Bendiksen E Brandrud TE Eidissen SE Lorås J Morozova O Dima B (2018) *Entoloma aurorae-borealis* sp. nov. and three rare *Entoloma* species in the Sinuatum clade (subg. Entoloma) from northern Europe. Sydowia 70: 199–210.

[B52] Peck CH (1902) Report of the State Botanist (1900). Annual Report on the New York State Museum of Natural History 54: 131–199.

[B53] Posada D Crandall KA (1998) Modeltest: Testing the model of DNA substitution. Bioinformatics (Oxford, England) 14(9): 817–818. 10.1093/bioinformatics/14.9.817 9918953

[B54] Reschke K Noordeloos ME Manz C Hofmann TA Rodriguez-Cedeño J Dima B Piepenbring M (2022a) Fungal diversity in the tropics: *Entoloma* spp. in Panama. Mycological Progress 21: 93–145. 10.1007/s11557-021-01752-2

[B55] Reschke K Morozova OV Dima B Cooper JA Corriol G Biketova AY Piepenbring M Noordeloos ME (2022b) Phylogeny, taxonomy, and character evolution in Entoloma subgenus Nolanea. Persoonia 49: 136–170. 10.3767/persoonia.2022.49.04 38234382 PMC10792224

[B56] Romagnesi H Gilles G (1979) Les Rhodophylles des forêts côtières du Gabon et de Côte d’Ivoire (Beihefte zur Nova Hedwigia, Vol. 59). Johann Christian Cramer Verlag, Stuttgart, 649 pp.

[B57] Sato H Satomi O Sugiyama Y (2025) Two new species of the *Entoloma quadratum-murrayi* complex from Japan, *E. kermesinum* sp. nov. and *E. flavescens* sp. nov. PLOS ONE 20(2): e0302695. 10.1371/journal.pone.0302695 PMC11819469 39937720

[B58] Selby PJ Babington CC Gray JE Henfrey A Francis A (1859) The annals of magazine of natural history, including zoology, botany and geology. Taylor and Francis, London, 472 pp.

[B59] Thompson JD Gibson TJ Plewniak F Jeanmougin F Higgins DG (1997) The Clustal-X windows interface: Flexible strategies for multiple sequence alignment aided by quality analysis tools. Nucleic Acids Research 63: 215–228. 10.1093/nar/25.24.4876 PMC147148 9396791

[B60] Vilgalys R Hester M (1990) Rapid Genetic Identiﬁcation and Mapping of Enzymatically Ampliﬁed Ribosomal DNA from Several Cryptococcus Species. Journal of Bacteriology 172: 4238–4246. 10.1128/jb.172.8.4238-4246.1990 2376561 PMC213247

[B61] Zhang D Gao F Jakovlić I Zou H Zhang J Li WX Wang GT (2020) PhyloSuite: An integrated and scalable desktop platform for streamlined molecular sequence data management and evolutionary phylogenetics studies. Molecular Ecology Resources 20(1): 348–355. 10.1111/1755-0998.13096 31599058

[B62] Zhong XR (2019) Phylogenetic and taxonomic studies on *Clitopilus* and its allied genera resources from China. Master’s Thesis, South China Agricultural University, Guangdong, China.

